# Assessment of the Impact of Shot-Peening on the Fatigue Life of a Compressor Blade Subjected to Resonance Vibrations

**DOI:** 10.3390/ma13245726

**Published:** 2020-12-15

**Authors:** Arkadiusz Bednarz, Wojciech Z. Misiolek

**Affiliations:** 1 Department of Aerospace Engineering, Rzeszow University of Technology, 35-959 Rzeszow, Poland; 2 Loewy Institute, Lehigh University, Bethlehem, PA 18015, USA; wzm2@lehigh.edu

**Keywords:** fatigue life, crack initiation, resonance vibration, shot-peening, residual stress

## Abstract

This publication presents an assessment of the influence of a surface treatment such as shot-peening on the fatigue life of a compressor blade exposed to resonant vibrations. As part of the work, a geometric model of the blade was developed, and a numerical modal and fatigue analysis were performed. The fatigue analysis was based on the Manson–Coffin–Basquin and Ramberg–Osgood models. Additionally, the location of the highest equivalent stresses was established. Based on the results of the strength analysis, two points were identified where a fatigue crack may potentially occur. As part of the work, the influence of different values of residual stresses on the results of the fatigue life was determined. The obtained results were compared to the literature values of fatigue life for this blade. A secondary objective of the study was to determine the size of the grains at various points of the blade, as well as the thickness of the layer plasticized as a result of peening. The relationship between the location of the highest values of the equivalent stresses and the thickness of the plasticized layer was determined. An explanation of the effect of shot-peening on the increase in the fatigue life of the blade was proposed.

## 1. Introduction

Compressor blades are often called the critical elements of the turbine aircraft engine. This statement is connected to the blade geometry, work conditions, and potential failures. Blades are thin in comparison with the rest of the geometrical parameters (height and length). This feature means that the blade is susceptible to resonant bending. On the other hand, the aero dynamical and centrifugal forces as a result of a high rotational velocity act on the examined blade [1,2]. The main function of the compressor is to create high pressure to allow energy transmitted by the blades to be transformed into kinetic energy for the working fluid. In the next components of the turbine engine, this air is mixed with fuel and is burned. The generated fumes drive the turbine vanes and also create the thrust of the engine.

The working compressor vanes create negative pressure for the air intake, which could be responsible for sucking small elements from the surroundings, such as small stones, grains of sand, and even birds, into the engine. The collision of the rotating blade with harder elements might create a notch on the blade surface [3,4,5]. If the notch is located close to the foot of the blade, it is usually the origin of a future crack. A damaged blade used in service could cause crack propagation and it could break at the feather. A broken off fragment of the blade may affect the balance of the compressor. An imbalance in the compressor could lead to resonance, which drastically would decrease the fatigue life of the whole engine.

The working conditions and possible damage of the blades are included in the design and manufacturing process. In the production of aircraft engine blades, special alloys (super alloys) are used. In order to create blades more resistant to foreign object damage (FOD) and crack initiation [6], the feathers are usually subjected to shot-peening, and special surfaces coatings are applied (e.g., to prevent corrosion).

Many papers dedicated to shot-peening and its impact on fatigue life are published in the literature. Shot-peening as a surface treatment has an astonishing impact on crack initiation and the overall fatigue life of the treated element. Shot-peening should be understood as a dynamic, plastic deformation surface treatment. The basis of this technology is to shoot small balls on the treated surface to create plastic deformation and, as a result, a compressive stress on the surface of the treated element.

Depending on the process parameters, it is possible to achieve residual stress with a value of −1200 MPa [7]. The distribution and levels of the residual stress are dependent on the kinetic energy and other parameters of shot-peening. The thickness of the plasticized layer may reach up to 100 μm (0.1 mm) [8]. Below this point, a quick increase in stress is observed, reaching values close to zero [9]. Additionally, the microhardness may increase even to 500 HV [10]. For example, Zhang [8] observed an increase in hardness equal to 60% (from 100 to value of 160 HV). 

Chengfang [11] observed that, in the case of a titanium alloy (super alloys) with residual stress equal to −750 MPa, the specimens achieved a 67 times higher fatigue life. On the other hand, Daoxia [7], in the case of residual stress equal to −1200 MPa for steel, found their specimens achieved 4.5 times greater fatigue life. Gao [12] showed that after shot-peening up to a residual stress level of −350 MPa, the specimens achieved a five-fold increase in fatigue life. Maleki [13] showed that, in case of residual stress equal to −650 MPa, in AISI 1045 specimens, a 10 times higher fatigue life was observed. Similar observations were made by Hammond [14], Seddik [15], and Dongxing [16]. The above-mentioned alloys are used as materials for aircraft rotors and for stationary flow machines (especially in the so-called cold zones). Based on the above analysis, it was found that there is no simple relationship between the level of initial/residual stress and change in fatigue life.

Tekeli, in his work [17], observed that in the case of brittle steel (SAE 9245), the increase in fatigue life was equal to 30% when the residual stress was equal to −480 MPa and the depth of the plasticized layer was equal to 33 μm.

The main goal of the present paper is to show the impact of shot-peening on the fatigue life of the compressor blade, under controlled geometry with a notch created by machining, subjected to resonance vibrations. The obtained results are used to explain the influence of the depth of the plasticized material layer on crack initiation and its propagation. The obtained results are of great importance for the safety of the air transport.

## 2. Numerical Analysis 

### 2.1. Analysis Assumptions

As part of the numerical analysis, a geometric model of the blade was built and the fatigue model of the EI-961 material (chemical composition: 0.1–0.16% C, 10.5–12.5% Cr, 0.35–0.5% Mo, 1.5–1.8% Ni, and 0.18–0.3% V) [18] for the ε-N analysis was developed. The geometric model included a geometric notch (no pre-stress). The blade was subjected to modal analysis in order to determine the stress distribution in the area of the notch during resonance bending. The information obtained from the performed analysis was used to determine the fatigue life of the tested blade. The obtained results were compared with the results for the tested blade published in the literature [3,5], made of from the same material—EI-961.

**Figure 1:**
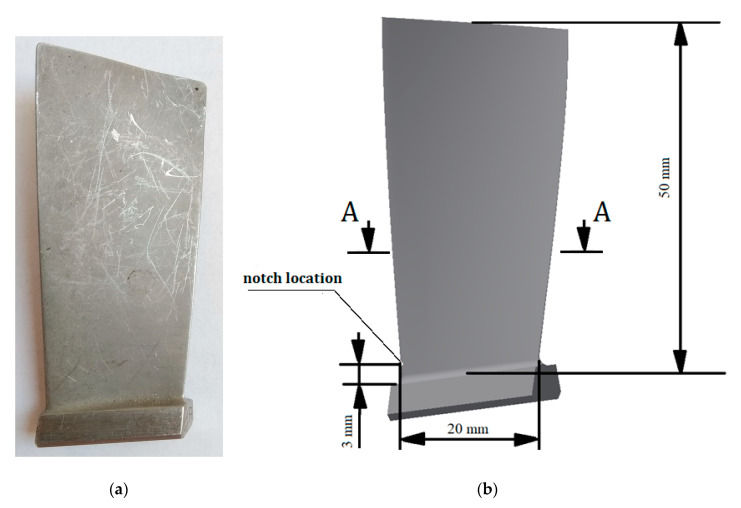
View of the (**a**) examined blade and (**b**) geometrical model used in the numerical analysis.

The object of our study was the first stage compressor blade of the PZL-10W turbine engine [3,4]. This blade (Figure 1a) is made of the alloy EI-961. It is an alloy with a Young’s modulus of E = 200 GPa, density ρ = 7800 kg/m^3^, tensile strength UTS = 1200 MPa, yield strength $\sigma_{Y}$ = 1000 MPa, and Poisson coefficient ϑ = 0.3 [5,18]. The aforementioned values are necessary in order to perform the numerical modal analysis, as well as to estimate the fatigue constants for this alloy.

**Figure 2:**
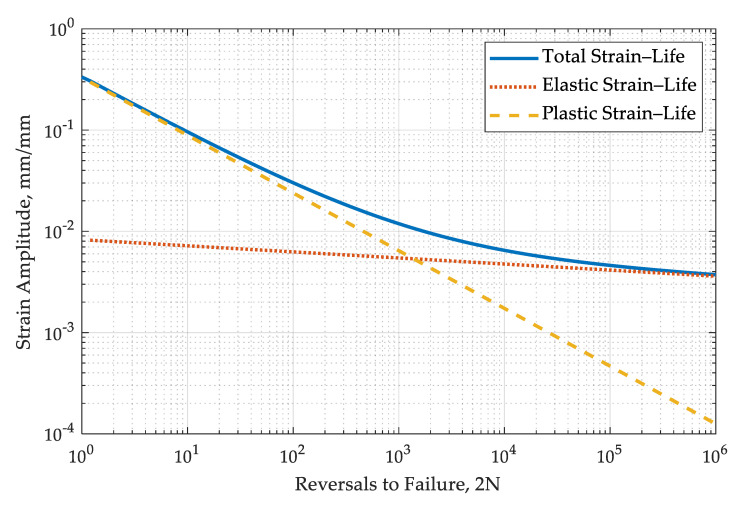
Strain–life curve for the EI-961 alloy (based on the Manson–Coffin–Basquin equation).

The performed fatigue analysis was based on the Manson–Coffin–Basquin Equation (1), result of which is the total strain amplitude, and the input data are the number of load cycles (N) and fatigue constants (Figure 2).

(1) $$\frac{\Delta \varepsilon }{2}=\frac{\sigma_{f}{}^{\prime }\;}{E}{\left(2N\right)}^{b}+\varepsilon_{f}{}^{\prime }{\left(2N\right)}^{c},$$

Within this model, it is necessary to estimate the following four material constants: $\sigma_{f}{}^{\prime }$, the fatigue strength coefficient; *b,* the fatigue strength exponent; $\varepsilon_{f}{}^{\prime }$, the fatigue ductility coefficient; and *c*, the fatigue ductility exponent. These values were taken from the literature [3] and were defined as the arithmetic mean value:$\;\sigma_{f}{}^{\prime }$ = 1651.94 MPa, *b* = −0.06, and $\varepsilon_{f}{}^{\prime }$ = 0.33, *c* = −0.57.

Because the modal analysis is based solely on the linear material model, and the EI-961 alloy is characterized by hardening, it is necessary to use the Ramberg–Osgood Equation (2) to determine the total deformation (elastic and plastic components).

(2) $$\Delta \varepsilon =\frac{\Delta \sigma }{E}+2{\left(\frac{\Delta \sigma }{2K{}^{\prime }}\right)}^{1/n{}^{\prime }},$$

To determine the strain amplitude (∆*ε*), it is necessary to estimate the following two values: $K{}^{\prime }$, the cyclic strength coefficient, and n′, the cyclic strain hardening exponent. These values were determined based on the data published in the literature [3], and are $K{}^{\prime }$ = 2053.27 MPa and n’ = 0.172.

The obtained data for the Manson–Coffin–Basquin equation and the Ramberg–Osgood equation were used to carry out the fatigue analysis based on the author’s (AB) calculation program.

### 2.2. Numerical Analysis

**Figure 3:**
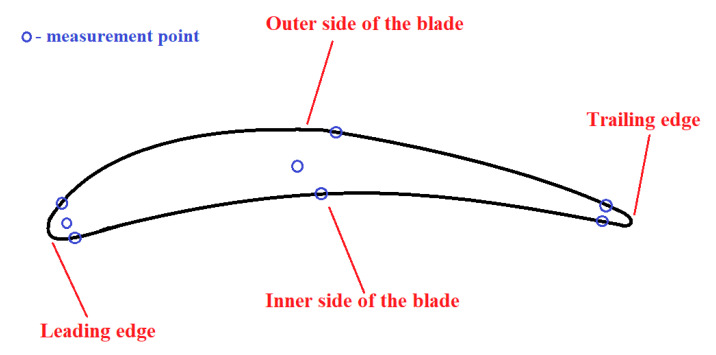
A sketch of a magnified cross-section with the marked measurement locations and geometrical terms for the analyzed blade.

**Figure 4:**
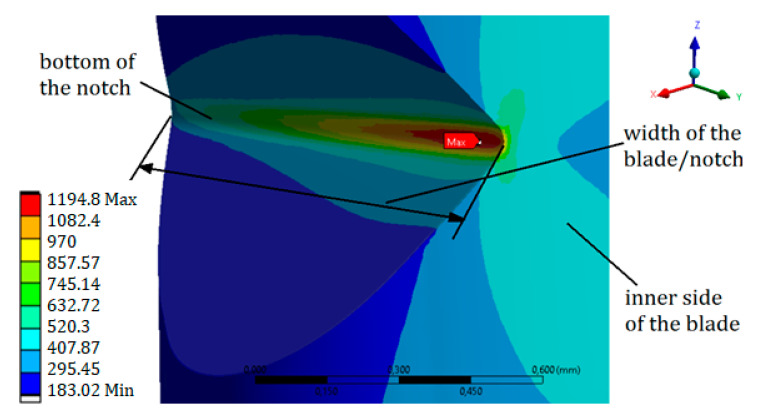
Equivalent stress $\sigma_{EQV}$ (MPa) distribution at the bottom of the notch, in the case of a resonance vibration with an amplitude equal to 1.8 mm.

Based on the real blade specimen dimensions, a geometric model was created. It included a geometric notch (V-type) that was 0.5 mm deep and located 3 mm from the foot of the blade (Figure 1b). The notch was located at the leading edge (Figure 1b and Figure 3). The rounding radius at the bottom of the notch was 0.05 mm. At the bottom of the notch, a width of 1.02 mm was observed (showed at Figure 4).

The geometric model of the notched blade was discretized with a finite element mesh, with a dimension not exceeding 0.01 mm in the bottom of the notch. The discrete model consisted of only tetrahedral elements (TET-10) with square shape functions. The prepared model was subjected to numerical modal analysis. The research was conducted using commercial ANSYS 19.2 software. The resonance frequency of the tested blade was 791.2 Hz. Experimental studies [3,5] showed that the tested element had the frequency of the first form of resonance vibrations in the range of 756 to 830 Hz.

**Figure 5:**
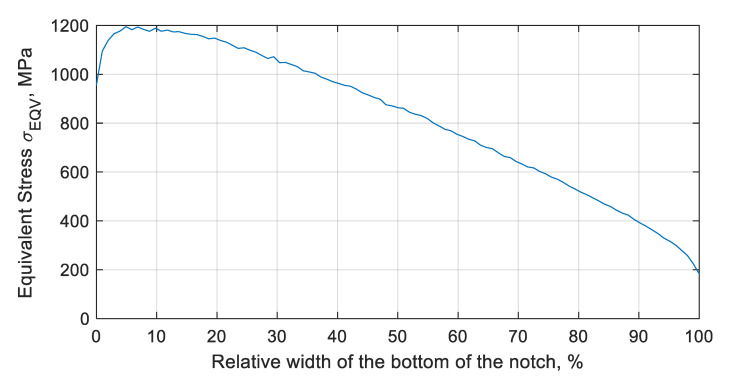
A plot of equivalent stress $\sigma_{EQV}$ (MPa) at the bottom of the notch, in the case of a resonance vibration with an amplitude equal to 1.8 mm.

During the resonant vibrations (with the first mode of free vibration), pure bending was observed. In the case of a vibration amplitude of 1.8 mm—measured as the displacement of the blade top—the stress concentration was recorded in the area of the notch bottom (Figure 4). In order to better visualize the results, a graph was prepared (Figure 5) showing the value of the equivalent stresses at the bottom of the notch, depending on the distance from the inner side of the blade, included in the relative values.

The observed equivalent stresses at the bottom were close to the value of the tensile strength (UTS) for this alloy. At the very edge of the element, the equivalent stresses reached a value slightly lower than the yield point ($\sigma_{Y}$), and amounted to $\sigma_{EQV}=955\;\mathrm{MPa}$ (yield strength $\sigma_{Y}$ = 1000 MPa). The maximum value of the equivalent stresses of $\sigma_{EQV}=1194\;\mathrm{MPa}$ was at the bottom of the notch, approximately 0.07 mm (70 µm) from the inner side of the blade. Thus, the maximum value of the stresses was not observed at the edge of the notch, but at the bottom. This, in turn, had a significant impact on the fatigue life. Taking into account the applied surface treatment (shot-peening), the initial/residual stress ratio from the blade surface (identical to the notch edge) significantly increased the fatigue life. The stress component (Z), related to the axis/height of the blade, had the greatest influence on the level of equivalent stress. 

In the next step, the calculated values of the equivalent stresses were used for the fatigue analysis, based on the previously mentioned Manson–Coffin–Basquin model. The performed ε-N analysis originally assumed a pendulum load cycle. In the subsequent iterations, different values of residual stresses were taken into account, from −100 to −500 MPa. Taking pre-stress values into account changed the load cycle to be two-sided with a negative average value. The results of the conducted analyses are summarized in Table 1 and Table 2.

In the first discussed case (when $\sigma_{EQV}=955\;\mathrm{MPa}$), assuming no residual stresses resulting from the surface treatment, only 117 load cycles were needed to initiate a crack (Table 1). In the case of residual stresses of −200 MPa, the number of cycles to crack initiation increased 16 times. A further increase in residual stress to −300 MPa caused an approximately nine-times increase in service life (to a value of $17\cdot 10^{3}$). 

In turn, in the case of a fatigue analysis based on the maximum value of the equivalent stresses ($\sigma_{EQV}=1194\;\mathrm{MPa}$), if the residual stresses were not taken into account, only 11 cycles were required to initiate a crack (Table 2). Only when the residual stresses of −500 MPa were taken into account was the observed durability at a level of $6.39\cdot 10^{3}$ load cycles.

In experimental studies [3], it was proven that a crack with a length of 0.2 mm was observed after about $12.9\cdot 10^{3}$ load cycles. Moreover, these tests show that the fracture started right at the bottom of the notch, not at its edge. As a result of the complex load condition in the bottom of the notch, as well as on the blade surface (residual stresses presence), it would be difficult to accurately estimate the number of cycles to initiate such a crack size.

## 3. Metallography

The next step of the research was the performance of the metallographic analysis, which resulted in information on the thickness of the layer exhibiting plastic deformation due to shot-peening, as well as the average grain size depending on the location within the blade (Figure 3).

**Figure 6:**
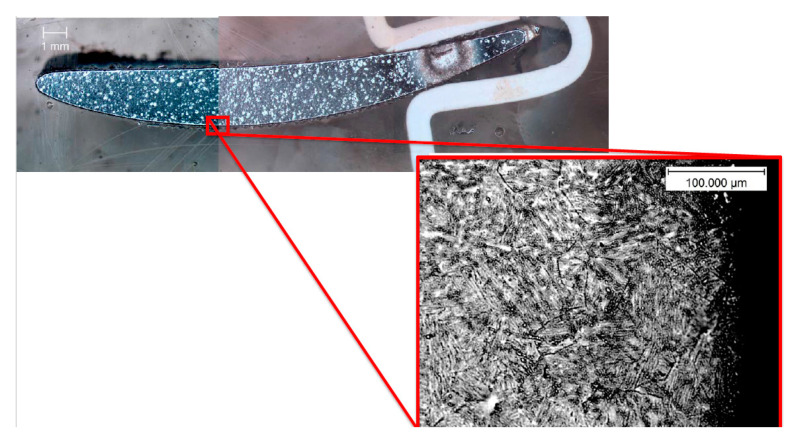
Picture of a cross-section of the blade with a magnified grain image from the center to the outer side of the blade.

**Figure 7:**
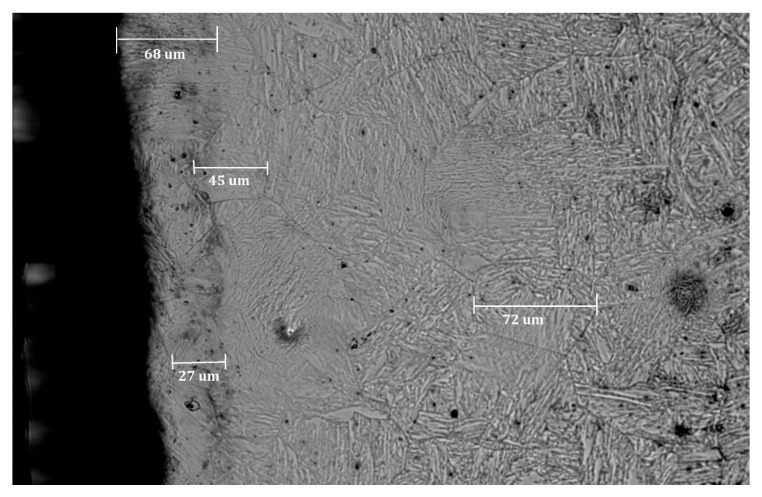
View of the microstructure of the seventh cross-section, on the inner side of the blade with marked representative dimensions (grain size and depth of the plasticized layer).

**Figure 8:**
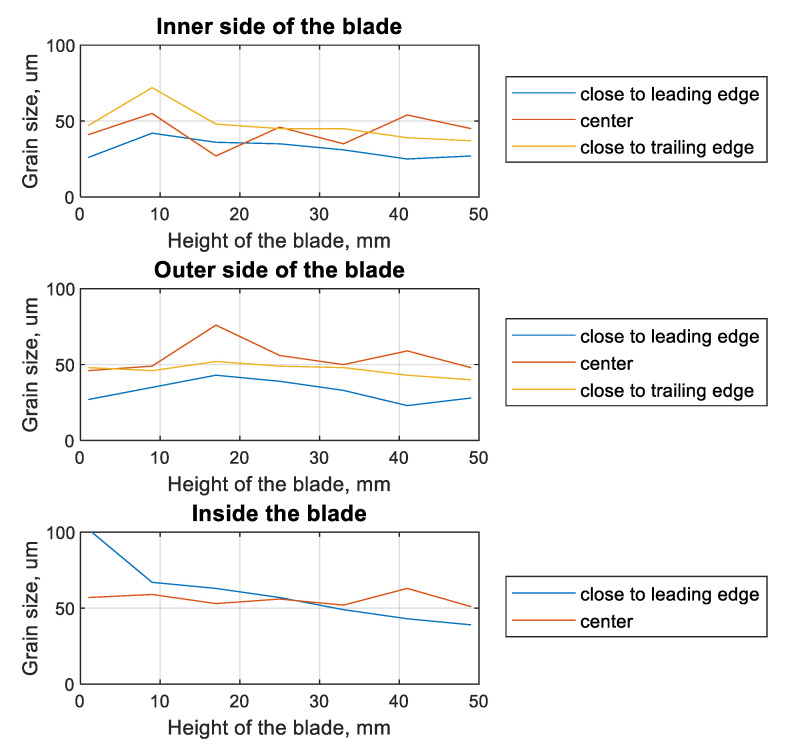
Plots of the grain size (in the layer plasticized by shot-peening) as a function of the height of the blade for different measurement locations.

**Figure 9:**
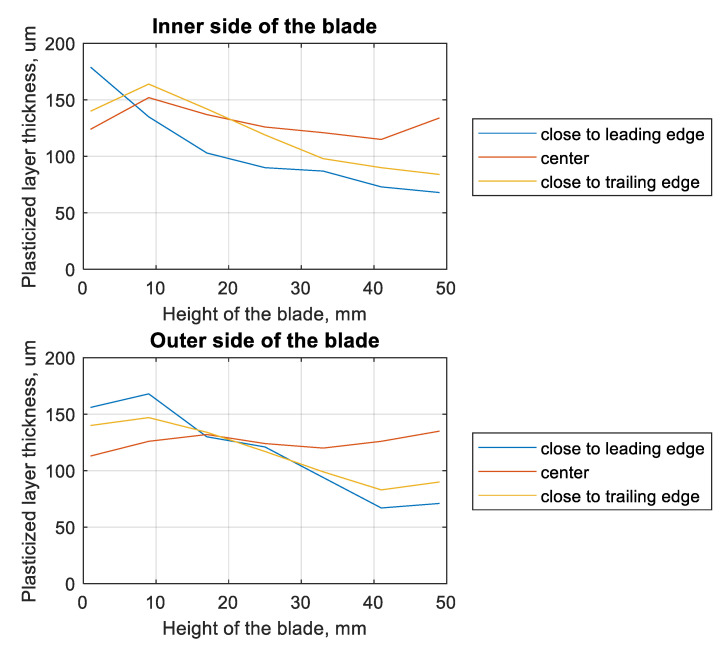
Plots of plasticized layer thickness (by shot-peening) as a function of the height of the blade for different measurement location.

**Figure 10:**
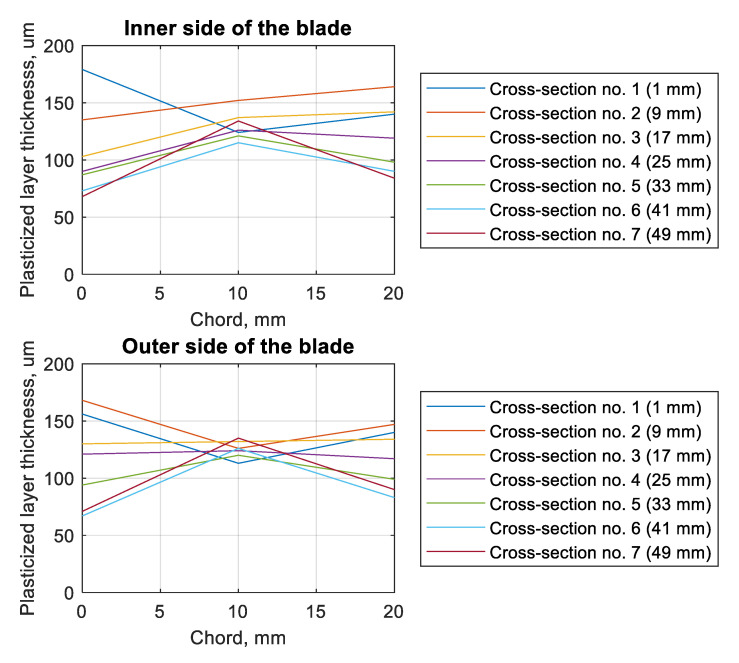
Plots of plasticized layer thickness (by shot-peening) as a function of the chord of the blade for a different side of the blade, for different cross-sections.

The compressor blade (Figure 1a) was cut with a diamond saw into 8-mm thick samples. These samples were mounted using a resin and were polished. In the last step, the samples were subjected to etching in order to emphasize the grain size and the presence of grain boundaries (Figure 6). The marble etchants (H_2_O, HCl, and CuSO_4_) were used while undergoing electrolysis etching for 20 s for each sample. The samples prepared with the typical metallography techniques were observed under an optical microscope. Using the image analysis software, a series of photographs were taken showing the grain size at different points within the blade profile (Figure 6). The size of a single grain was measured, as well as the thickness of the layer plasticized as a result of surface treatment—shot-peening (Figure 7). The measurement was made on both sides of the blade profile, at six characteristic points (at the leading edge, in the middle part, and at the trailing edge—on the inner and outer sides of the profile), as well as at two points inside the profile. The locations of the points are presented in Figure 3. It should be remembered that the inner side of the blade is the working side that is most exposed to erosive action, and that the leading edge is most exposed to collision with hard elements sucked into the engine. The obtained grain measurement results are presented in Figure 8, Figure 9 and Figure 10.

As shown in Figure 7, it was possible to measure the size of the grains and the thickness of the plasticized layer using an optical microscope with a magnification up to 1000×. The grain size measurements were based on the ASTM 112-13 standard (intercept procedure). A distinctive boundary between the plasticized zone and the non-plasticized zone was present. The representative grain size of the native material was 72 µm, while at the edge the grain size was about 27 µm. The thickness of the plasticized layer in this part of the blade was 68 µm. In the presented cross-section, a smaller thickness of the plasticized zone by peening was observed.

As a result of the performed work, the smallest grains were observed on the inner side of the blade (Figure 8), at the leading edge (26 µm), while the largest were observed at the trailing edge of the profile—47 µm). This tendency continued until about half the height of the blade. At the tip of the blade, the largest grains were observed in the middle (48 µm) of the inner side of the blade. In the case of the outer side, the smallest grains were observed near the leading edge (27 µm), and the largest (up to three times larger) in the middle part of the profile (46 µm). In the case of the native material (inner side of the blade), there was a tendency that in the part at the leading edge, the grains were almost two times larger than in the central part. Generally, the grains were 25 to 72 µm on the inner side and 23 to 76 µm on the outer side. In the case of the native material in the leading-edge zone, the grains ranged in size from 102 µm (at the root) to 39 µm at the tip of the blade. 

The distance from the root of the blade also affects the thickness of the plasticized layer (Figure 9). For the leading-edge zone, on both the inside and outside of the blade, a layer thickness of more than 150 µm was observed. For the central part of the blade as well as the outer side, the plasticized layer exceeded 100 µm, but did not exceed 150 µm. The greater the distance from the foot of the blade, the more significantly the thickness of the plasticized layer at the leading edge decreased. At the tip of the blade, the smallest thickness of this layer was recorded for the leading edge. The layer thickness in the trailing edge part was not much greater.

The change in the thickness of the plasticized layer was also verified depending on the chord of the blade and the tested cross-section (Figure 10). In parentheses, there is information about the distance between the foot of the blade and the given parallel cross-section. On both the inside and outside of the blade, in the central part of the blade, the smallest scatter in the obtained results was observed. In the case of the edges, the trends described in the discussion of Figure 9 were observed. In the case of cross-sections 1 and 2 (on the outer side of the blade), it was observed that the edges of the blade had a greater thickness for the plasticized layer. This tendency was not observed in the remaining cross-sections. 

The tested blade had not been used before testing and analysis, and the observed fatigue life corresponded to approximately 216 min of operation in the resonance state. Taking into account the fact that if the duration of a typical flight exceeds 3 h, there is a risk of an early rupture of the blade in the event of damage and a simultaneous resonance state. The location of the notch also affects the possibility of blade breaking. The further away from the root of the blade the notch is located, the less chance of the blade breaking and the lower the possibility of serious damage to the jet engine and the aircraft. This possibility was associated with both a reduction in the stresses in the resonant state, as well as a small decrease in the thickness of the deformed zone of the blade due to applied surface treatment.

## 4. Conclusions

The obtained results of the numerical fatigue analysis show that during the vibration of the compressor blade with a geometric notch, stresses occur close to the tensile strength for a given alloy. The classic approach related to the determination of the fatigue life using the Manson–Coffin–Basquin model does not give satisfactory results. Only taking into account the values of initial/residual stresses resulting from the surface treatment allows for obtaining more accurate results. Experimental studies related to the determination of the thickness of the plasticized layer show that the area of maximum reduced stress values is contained in this zone. Thanks to this approach, it is possible to increase the durability of the blade.

The obtained results can also be used to assess the influence of a different notch location on the fatigue life. The thicker the layer of the plasticized zone and the higher the values of residual stresses, the higher the fatigue life. The observed decrease in the thickness of the plasticized zone near the leading edge may contribute to a decrease in fatigue life (in the case of observation of the notch on a higher distance from the foot of the blade). Similar observations can be made for the thickness distribution along the chord of the blade. The thicker the plasticized zone, the slower the fatigue crack development process, which translates directly into the number of load cycles necessary to break the blade.

Future research should focus on the accurate determination of the initial stress values on the blade surface, as well as the optimized fatigue data determination method for numerical fatigue analysis.

## Figures and Tables

**Table 1:** Results of the numerical fatigue analysis based on the equivalent stress at the notched edge, with an amplitude of resonant vibrations of 1.8 mm.

	Assumed Value of Residual/Initial Stresses Created by Shot-Peening					
	0 MPa	−100 MPa	−200 MPa	−300 MPa	−400 MPa	−500 MPa
**Eqv. Stress on the Edge of the Notch** $\sigma_{EQV}=955\;MPa$	117	405	$1.89\cdot 10^{3}$	$17\cdot 10^{3}$	$875\cdot 10^{3}$	$259\cdot 10^{6}$

**Table 2:** Results of the numerical fatigue analysis based on the maximum value of the equivalent stress, with amplitude of resonance vibrations of 1.8 mm.

	Assumed Value of Residual/Initial Stresses Created by Shot-Peening					
	0 MPa	−100 MPa	−200 MPa	−300 MPa	−400 MPa	−500 MPa
**Max. Value of Eqv. Stress** $\sigma_{EQV}=1194\;MPa$	11	28	76	243	987	$6.39\cdot 10^{3}$
